# Cytology Techniques Can Provide Insight into Human Placental Structure Including Syncytiotrophoblast Nuclear Spatial Organisation

**DOI:** 10.3390/jdb11040046

**Published:** 2023-12-15

**Authors:** Cassie Fives, André Toulouse, Louise Kenny, Therese Brosnan, Julie McCarthy, Brendan Fitzgerald

**Affiliations:** 1 Department of Pathology, Cork University Hospital, T12 DC4A Cork, Ireland; 2 Department of Anatomy and Neuroscience, University College Cork, T12 XF62 Cork, Ireland; 3 Faculty of Health and Life Sciences, University of Liverpool, Liverpool L69 7TX, UK; 4 Pregnancy Loss Research Group, Department of Obstetrics and Gynaecology, University College Cork, T12 YE02 Cork, Ireland

**Keywords:** placenta, cytology, placental cytology, fine-needle aspiration, syncytiotrophoblast, trophoblast nuclear organisation patterns, linear nuclear arrangements, syncytial knots, trophoblast turnover

## Abstract

The aim of this study was to provide the first systematic description of human placental cytology appearances and to investigate syncytiotrophoblast nuclear organisation patterns using cytology techniques. Term placentas from normal pregnancies were sampled using fine-needle aspiration (FNA) and direct scrapes. Standard histological examination was also performed to exclude pathological changes in the placentas being studied. Both Papanicolaou-stained cytospin preparations and air-dried Giemsa slides from FNA provided high-quality material for cytological assessment with good cellularity. Among the key features of the cytology preparations were villous “microbiopsies” that allowed for the three-dimensional appreciation of villous branching patterns. Cytological appearances, including nuclear characteristics of villous cytotrophoblast and syncytiotrophoblast, were also well demonstrated. In microbiopsies and detached villous trophoblast sheets, complex patterns of syncytiotrophoblast nuclear organisation, not previously described cytologically, were observed, including irregular spacing of nuclei, syncytioplasm windows and linear nuclear arrangements. This study showed that placental cytology (a) provides technically excellent material for cytological evaluation, (b) confirms the presence of complex nuclear organisational patterns in the syncytiotrophoblast by eliminating the possibility of tangential sectioning artefact, (c) provides superior nuclear detail over standard histological sections and (d) may be an untapped research resource for the investigation of normal and pathological processes because of its ability to look at the placenta in a novel way and through its potential for both ex vivo and in vivo placental sampling.

## 1. Introduction

In the earliest stages of pregnancy, the placental structures begin to form as the blastocyst implants in the endometrium. Cells in the outermost epithelial layer of the blastocyst, the trophoectoderm, begin to fuse to form a primary syncytium with the resulting syncytiotrophoblast masses eroding into the endometrial glands, whose secretions provide nutrients to the developing pregnancy. Trophoectoderm cells that do not fuse to form syncytiotrophoblast are referred to as cytotrophoblast. They proliferate and push into the syncytium to form primary villi. While the trophoectoderm provides the future epithelial elements of the placenta, the extraembryonic mesenchyme extends into the primary villi at approximately 17 to 18 days post fertilisation to form the mesenchymal component and secondary villi; the basic arrangement of the placental villi is then in place [1]. Due to its critical role in implantation, differentiation of trophoectoderm proceeds even before significant embryonic development, emphasising the importance of even the earliest phases of placental differentiation and development [2].

Therefore, although we think of the placenta as separate to the embryo or foetus, their development is intrinsically linked even in the earliest stages of pregnancy. The placenta is in fact genetically and functionally an embryonic/foetal organ. In later pregnancy, it receives between 21% and 32% of the foetal cardiac output, depending on gestational age, and is responsible for oxygen and nutrient transfer to the developing foetus, waste product elimination and endocrine functions that are all essential to a successful pregnancy [3,4]. Beyond implantation, the continuous growth and functional development of this foetal organ is as important to long-term embryonic and foetal survival as that of any other critical structure, such as the heart. As an example, the development of the placenta and heart, as two of the earliest organs to form, are even thought to influence one another during subsequent pregnancy with associations between placental abnormalities and heart defects having been proposed or demonstrated [5]. It is also now recognised that in utero conditions have effects on foetal development that have long-term health consequences in the both the cardiovascular system and many other organ systems, including the renal, hepatic, pulmonary and central nervous system [6]. Thus, placental health is not just important solely for the outcome of a healthy, living neonate but for helping that baby to also become a healthy adult. Although the placenta is a temporary organ, its influence lingers throughout life.

We have discussed how placental development and health may have long-term health implications for the developing foetus. During the course of the actual pregnancy itself, however, the focus on the placenta is often because of its association with pregnancy complications, including stillbirth [7,8]. Apart from pregnancy loss, a placental origin has been implicated in major pregnancy complications, such as preeclampsia and intrauterine growth restriction (IUGR). More specifically, these latter complications have been linked to suggested abnormalities in placental trophoblast [9,10,11,12,13,14]. The trophoblast component of mature placental villi consists of cytotrophoblast and the overlying syncytiotrophoblast. As cytotrophoblast proliferates, some of the cells produced differentiate to fuse with the overlying syncytiotrophoblast to allow it to be replenished and grow [15]. Syncytiotrophoblast, which covers the outermost aspect of placental villi, is unusual in the human body in that it is a syncytium within which nuclei are dispersed without dividing cell membranes. Different fragments and particles of syncytiotrophoblast are shed into the maternal circulation during pregnancy so that an element of continuous turnover exists within the syncytiotrophoblast compartment [15].

The organisation of nuclei in linear arrangements within syncytiotrophoblast has been previously described and proposed as a feature underlying a distinctive pattern of pathological syncytial knot formation within the placenta, known as wave-like syncytial knots (WLSK) [16]. We hypothesised that these linear nuclear arrangements must have a function within the syncytium because for a syncytium like the syncytiotrophoblast to function efficiently, the spatial organisation of its components, including nuclei, must be regulated in some manner, potentially from the earliest stages of its formation. Similarly, the normal process of shedding of syncytiotrophoblast nuclei, and therefore villous trophoblast turnover in general, must also include a degree of spatial coordination, but little is known about this potential trophoblast characteristic. There is precedent for this concept in another multinucleated structure, the skeletal muscle myofibre. In skeletal muscle, complex nuclear movements and positioning occur during myofibre formation; nuclear clustering also occurs under the neuromuscular junction in a process that demonstrates how nuclear spatial positioning links with function [17]. In syncytiotrophoblast, the potential for such spatial organisation, nuclear movement and links with trophoblast function is largely unexplored.

Various techniques have been used to investigate placental appearance and structure, including standard histology, stereology and electron microscopy. As yet, to our knowledge, modern cytology techniques have not been applied. One tool with potential for investigating trophoblast nuclear organisation is cytology, as cytology preparations eliminate the possibility of tangential artifacts that have complicated the evaluation of placental structure in the past when standard histological sections were used.

The aims of this study were therefore twofold: (1) to describe for the first time the appearances of the normal term human placenta using modern cytological techniques and (2) to determine whether cytology is a useful tool for investigating syncytiotrophoblast nuclear organisation patterns.

## 2. Materials and Methods

**Figure 1:**
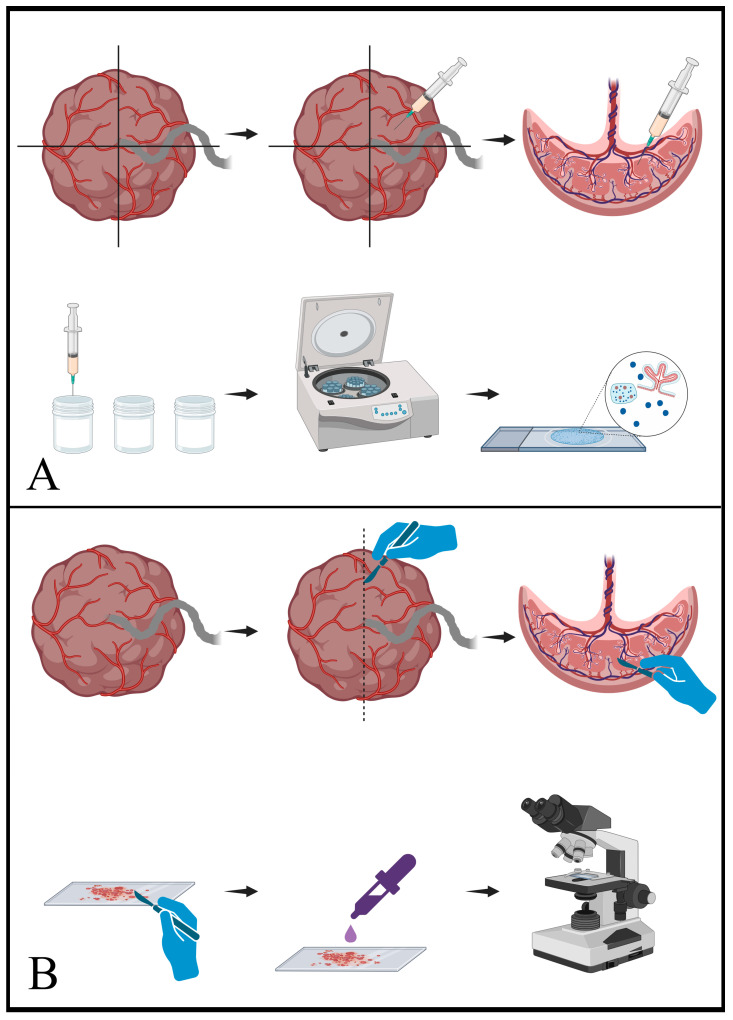
Schematic representation of cytology sampling techniques. (**A**) shows the procedure for fine-needle aspiration (FNA). In the upper sequence, a freshly delivered placenta is arbitrarily divided into quadrants and a sampling needle is passed through the foetal surface (chorionic plate) into the parenchyma. Negative pressure applied from the syringe draws fragments of the parenchyma into the needle and syringe. In the lower sequence, the material from the syringe and needle is transferred into vials of either CytoLyt or normal saline. This material is then centrifuged and processed to generate the final slides, which contain a mixture of parenchymal elements. (**B**) shows the equivalent process for scrape samples. As seen in the upper sequence, a fresh placenta is sliced to reveal the cut surface of the placental parenchyma. A scalpel blade is then scraped along the cut surface and collects fragments of the parenchyma. In the lower sequence, the material present on the scalpel blade is transferred to a slide and then stained for microscopic interpretation. [Schematics created with BioRender.com (accessed on 7 December 2023)].

Within 30 min of delivery, fresh placentas were collected from clinically normal term pregnancies. Exclusion criteria included pregnancy-induced hypertension, gestational diabetes, preeclampsia and intrauterine growth restriction. The placentas were arbitrarily divided into four quadrants. Fine-needle aspiration was performed using the standard technique with a Cameco needle holder (SW18 4JQ, Cameco Ltd., London, UK) through the foetal aspect of the disc using 23-gauge needles. Three passes were taken per quadrant; two were taken into separate vials of CytoLyt (ThinPrep^TM^, CytoLyt^TM^, Hologic, Marlborough, MA, USA) for the generation of Papanicolaou-stained ThinPrep and cytospin preparations and one was taken into normal saline for the generation of May–Grünwald–Giemsa (MGG)-stained cytospins (Figure 1A) (Papanicoloaou stains from Leica Biosystems, IL, USA; MGG stains from Diapath, Martinengo, Italy). The placenta was then sectioned centrally and a scrape sample was taken with a scalpel blade from the central portion of the disc, spread on a glass slide, rapidly air-dried with a hair dryer and stained with MGG (Figure 1B). The placentas were then formalin-fixed and standard histological examination was performed for histological/cytological correlation and particularly to exclude significant developmental, inflammatory or destructive processes. Cytological preparations were reviewed by a cytopathologist and histological sections by a perinatal pathologist. A database of images of each observed cytological feature was created for documentation and repeated review.

### 2.1. FNA Cytology Slide Preparation Techniques

After FNA sampling, as outlined above, 3 separate sample vials were available per placental quadrant (1 in saline, 2 in CytoLyt). These vials were spun in a multispeed centrifuge at 2499 rpm for 5 min. The supernatant was then decanted and discarded and the cell pellets were vortexed (to liquefy them).

### 2.2. Cytospin

One drop of the liquid cell pellet from a CytoLyt sample and a saline sample were pipetted into separate cytofuge concentrator wells and spun in the cytocentrifuge at 1000 rpm for 4 min. This process transferred the sample material onto a glass slide, creating a “cytospin” slide. The cytospin slide generated from the CytoLyt sample was placed into 95% industrial methylated spirits for 20 min prior to staining with Papanicolaou. The cytospin slide generated from the saline sample was allowed to air dry prior to staining with MGG.

### 2.3. ThinPrep^®^

A drop of the liquid cell pellet from a CytoLyt sample was transferred to a ThinPrep^TM^PreservCyt^TM^ vial (Hologic, Marlborough, MA, USA). ThinPrep slides were generated using the ThinPrep®2000 processor (Hologic, Marlborough, MA, USA) according to the manufacturer’s protocol. Slides were stained with Papanicolaou.

### 2.4. Confocal Microscopy

One drop of the liquid cell pellet from a CyoLyt sample was resuspended in formalin for 30 min. The specimen was then centrifuged again at 2499 rpm for 5 min. The formalin supernatant was decanted and cytospin slides were made from the cell pellet as per the cytopsin method outlined above. These slides were placed in phosphate buffer saline and subsequently stained with bisbenzimide (10 µg/mL) for 3 min and mounted in FluorG medium. Image stacks (2 µm apart) were acquired on an Olympus FV1000 confocal microscope (Olympus UK and Ireland, Southend-on-Sea, UK).

### 2.5. Cell Block

One to two drops of human plasma and the same amount of Innovin (Dade®Innovin®, SiemensHealthineers, 90152, Erlangen, Germany) were added to the remaining cell pellets and allowed to stand for 1–2 min to allow clot formation. The clot was then fixed in 10% neutral buffered formalin for 24 h and then processed as per standard laboratory biopsy technique.

## 3. Results

Our study group comprised of placentas from eight patients from which 131 cytology slides were generated for evaluation. The mean gestational age at delivery was 38 weeks 3 days (range 37 weeks 2 days–39 weeks 4 days), and the mean birthweight at delivery was 3.4 kg (range 2.5–4 kg). The mode of delivery in seven cases was lower segment caesarean section and there was one vaginal delivery. There were no placental delivery complications. The average Apgar score at 5 min was 9. As per our exclusion criteria, there was no maternal pregnancy-induced hypertension, no intrauterine growth restriction and no preeclampsia. There was one maternal appendicectomy during pregnancy and there were no other pregnancy complications. Where relevant, there were no previous pregnancy complications.

**Figure 2:**
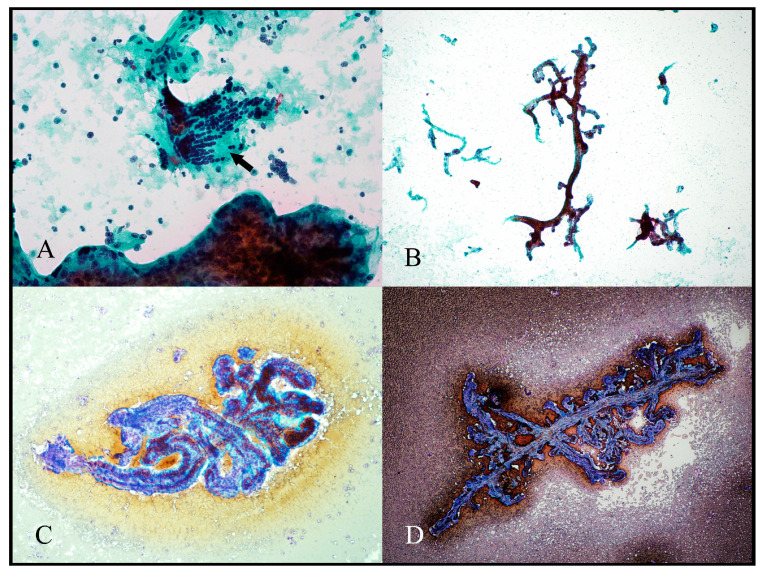
Microbiopsies. (**A**) shows the typical mixture of material seen in placental cytology samples with a microbiopsy in the lower part of the field, multiple single nuclei scattered throughout and a detached sheet of villous trophoblast cells (arrow). (**B**–**D**) show various microbiopsies clearly showing the branching nature of the villous tree fragments. [(**A**) Papanicolaou cytospin, 400×; (**B**) Papanicolaou cytospin, 40×; (**C**) May–Grünwald–Giemsa (MGG) cytospin, 100×; (**D**) MGG scrape, 40×].

All preparations and all stains provided abundant cytological material for evaluation. On low power, the most striking architectural feature was the presence of microbiopsies showing the arborising branching nature of the chorionic villi (Figure 2). These microbiopsies were present on all preparations but were most abundant on cytospin preparations, where they remained intact; they were least abundant on ThinPrep. Microbiopsies were best observed in MGG-stained preparations (Figure 2C,D). The background within slides was composed of blood with a few dissociated cells on air-dried smears and scattered dissociated cells on ThinPrep slides.

**Figure 3:**
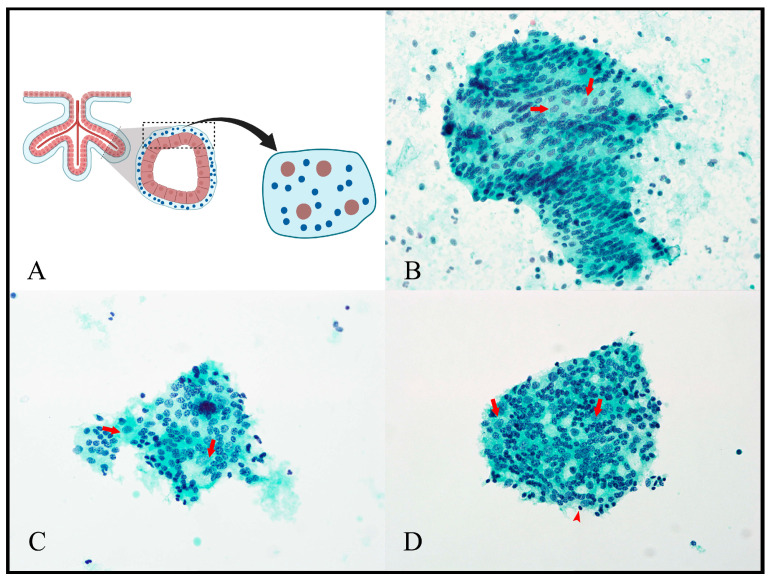
Trophoblast sheets. (**A**) shows a schematic representation of the origin of trophoblast sheets. The FNA or scraping process traumatises the villous tree and cleaves sections of villous trophoblast from villi between the cytotrophoblast and basement membrane layers. In the cytology preparations, these detached fragments of trophoblast appear as flat sheets. (**B**–**D**) all show examples of detached sheets of villous trophoblast. Cytotrophoblast nuclei have large nuclei and finely clumped chromatin and appear to sit in syncytioplasm “windows” (arrows). Syncytiotrophoblast nuclei are smaller and show increasingly clumped chromatin. Some of the syncytiotrophoblast nuclei are completely condensed (arrowhead in (**D**)). [(**A**) Created with BioRender.com (accessed on 7 December 2023). (**B**,**C**) Papanicolaou cytospins, 400×; (**D**) Papanicolaou ThinPrep, 400×].

**Figure 4:**
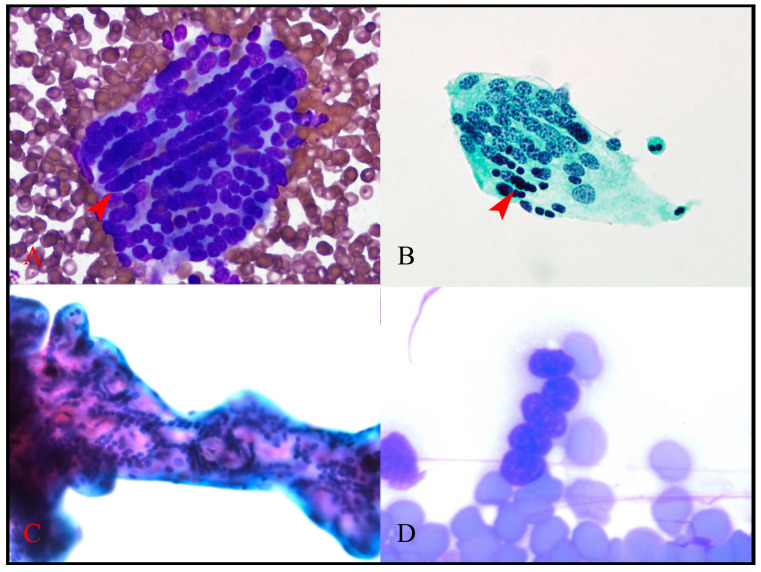
Nuclear arrangements. Complex patterns of syncytiotrophoblast nuclear organisation are observed. In (**A**), the distinctive linear arrangement of nuclei is demonstrated (arrowhead). In (**B**), linear arrangements are also observed, with condensed nuclei evident as a group within a line (arrowhead). In (**C**), the arrangement of nuclei within the syncytioplasm is observed in situ within a microbiopsy. In (**D**), naked nuclei devoid of syncytioplasm maintain their linear arrangement, suggesting binding to cytoskeletal elements or to one another. (**A**) MGG scrape, 400×; (**B**) Papanicolaou ThinPrep, 400×; (**C**) MGG scrape, 400×; (**D**) MGG scrape, 630×.

As well as microbiopsies, flat sheets of detached villous trophoblast were observed, providing clear views of this placental component. Complex patterns of syncytiotrophoblast nuclear arrangements, including irregular nuclear spacing and linear nuclear arrangements, were easily seen (Figure 3 and Figure 4). While these patterns were readily identifiable in flat trophoblast sheets (Figure 3), they could also be evaluated “in situ” in the microbiopsies by focusing through the intact villus structures (Figure 4C). This had the advantage of showing the relationship of nuclear arrangements to the axis of the villus and to villus branching points. In the slide background, “naked” syncytiotrophoblast nuclei, which were devoid of syncytioplasm but still maintained their linear nuclear arrangements, were identified (Figure 4D).

The nuclear chromatin pattern of the trophoblast nuclei appeared finely clumped in all preparations in all specimens. This was easiest to observe in the flat, detached villous trophoblast sheets and best seen in Papanicolaou-stained preparations (Figure 3). The chromatin patterns of the syncytiotrophoblast nuclei varied from finely clumped to completely condensed (Figure 4B).

Cytotrophoblast and syncytiotrophoblast nuclei were easy to distinguish, with cytotrophoblast nuclei appearing larger, paler and with more finely clumped chromatin (Figure 3). In the flat trophoblast sheets, the large cytotrophoblast nuclei were identifiable in window-like spaces between the syncytiotrophoblast nuclei (Figure 3).

**Figure 5:**
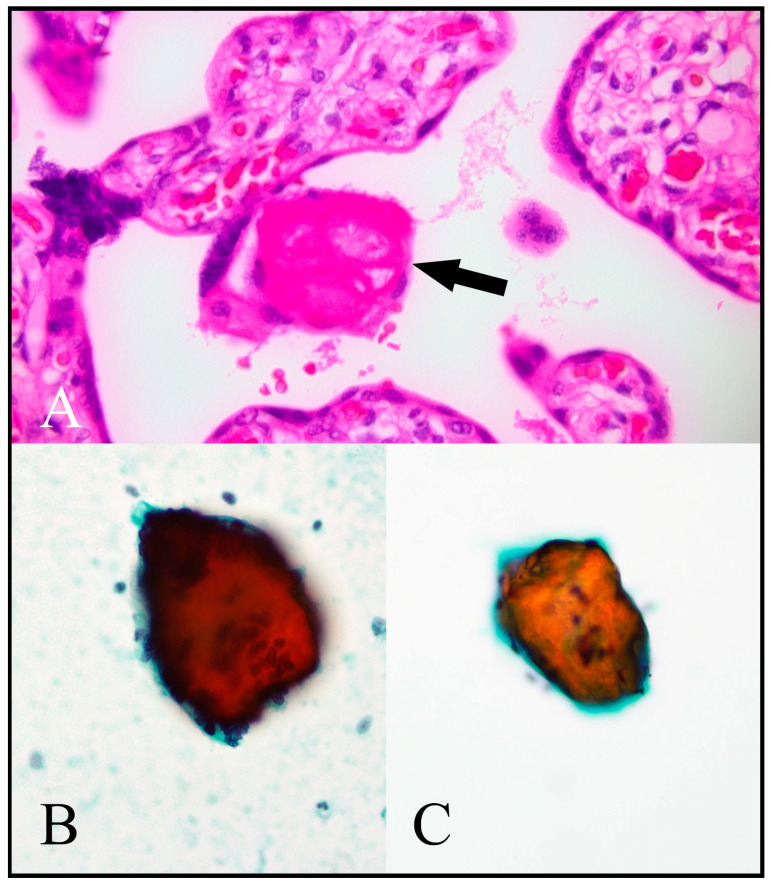
Fibrinoid. (**A**) A standard haematoxylin and eosin (H/E)-stained placental histological section shows a deposit of fibrinoid material (arrow). (**B**,**C**) show fibrinoid material in Papanicolaou-stained cytology preparations represented by orangeophilic crystalline fragments. [(**A**) H/E, 400×; (**B**) Papanicolaou cytospin, 400×; (**C**) Papanicolaou ThinPrep, 400×.].

Stromal and vascular components were easiest to appreciate in cell block preparations. Fibrinoid was easiest to appreciate in Papanicolaou-stained preparations, appearing as dense orangeophilic clumps (Figure 5).

**Figure 6:**
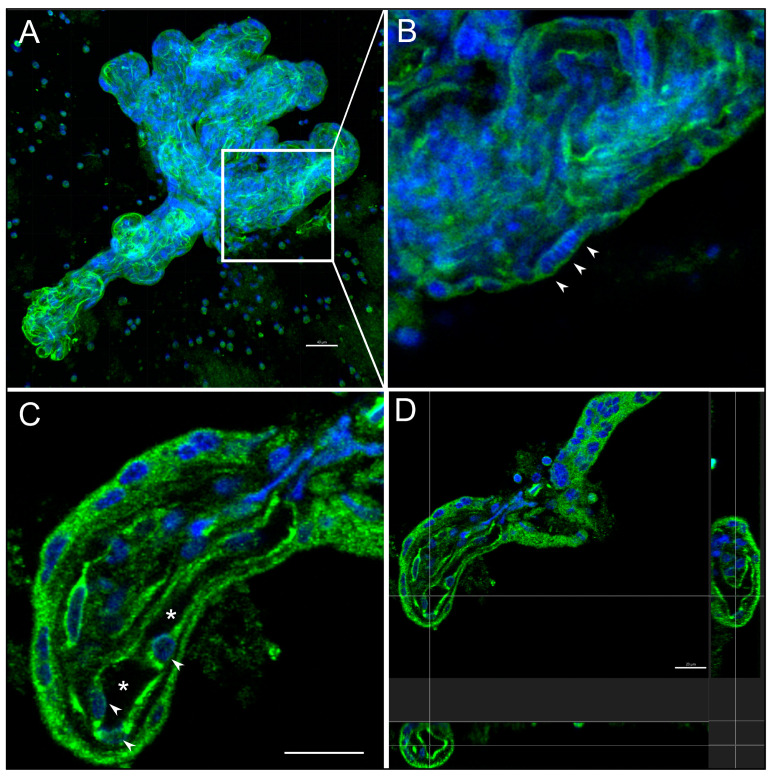
Confocal microscopy assessment of villous structure from cytospin slides. (**A**) Complex branching placental villous structure reconstructed from a stack of 12 confocal microscope images (2 µm thick). The presence of capillaries is revealed by green autofluorescence, while nuclear DNA is counterstained with bisbenzimide (20× magnification, scale bar = 40 µm). The white square indicates the region magnified in (**B**). (**B**) Single-image analysis from the stack reconstructed in A shows closely aggregated syncytiotrophoblast nuclei (arrowheads). (**C**) Single-image analysis reveals the presence of large capillaries (asterisk) and reveals details of the fine structure, including the presence of endothelial nuclei (arrowheads, scale bar = 20 µm). (**D**) 3D reconstruction and planar sections of a single villus reveal the complexity of the capillary shapes/arrangements (scale bar = 20 µm).

Microbiopsies present in cytospin material were processed for laser scanning confocal microscopy. Complex and simple villous structures were reconstructed in three dimensions from confocal microscopy stacks (Figure 6A). Image analysis revealed that villous capillaries were visible in autofluorescence (Figure 6A), while analysis of individual images from the stack (2 µm thick) allowed for the identification of closely aggregated nuclei similar to those observed in light microscopy (Figure 6B). Villous capillary lumens could be identified with endothelial cell nuclei in their walls (Figure 6C). Multiplane visualisation allowed for analysis of villous morphology, including volume reconstruction and location of capillaries (Figure 6D).

## 4. Discussion

For the first time, this study has documented the normal cytological appearances of the various components of the human placenta, with particular success in demonstrating cytological appearances of villous trophoblast, including details of nuclear morphology and syncytiotrophoblast nuclear spatial arrangements. To visualise nuclear arrangements within syncytiotrophoblast in a standard histological section, the section has to fortuitously pass through the thin layer of syncytiotrophoblast, parallel to its surface, over a distance sufficient to demonstrate the arrangements. Given the complex branching nature of the human placenta, such events are rare, with the result that the arrangements were only relatively recently described [16]. We have shown that the procedures involved in taking the cytology samples traumatise the villi so that sheets of trophoblast are detached and that these can be easily identified in the resultant preparations. The ability to visualise detached flat sheets of villous trophoblast is a unique feature of cytology preparations that cannot be replicated using standard histological sections. The ability to obtain placental microbiopsies also provided an opportunity to observe the three-dimensional structure of the terminal villous tree and the in situ appearance of syncytiotrophoblast nuclear arrangements in a manner also not possible in standard histological sections.

While links are established between placental abnormalities and pregnancy complications, methods of diagnosing and treating such conditions are still evolving. Sadly, some placental conditions are only diagnosed following an adverse pregnancy outcome or pregnancy loss and there are currently few methods of accurately diagnosing these conditions antenatally. The potential diagnostic role of placental FNA cytology is unknown, but in principle, we have shown that many diagnostic requirements could be met as placental components are presented in a variety of forms for interpretation. If FNA were to be used in making antenatal placental diagnoses, limitations in the volume of material that can be obtained would dictate that it would only be applicable for conditions that would affect the placenta diffusely, such as distal villous immaturity (DVI), chronic histiocytic intervillositis (CHI) and diffuse, high-grade chronic villitis of unknown aetiology (CVUA). Extensive ex vivo work would be required beforehand. While there would be reluctance to sample a placenta in vivo using FNA in the second and third trimesters, placental biopsy has previously been used to determine foetal karyotype in a study which performed transabdominal placental biopsy on 57 patients between 16 and 36 weeks of gestation, using needles with an external diameter of 1.5 mm without serious adverse events [18]. A further study compared second-trimester placental biopsy versus amniocentesis for prenatal diagnosis of β thalassemia. In this study, 181 placental biopsies were performed between 13 and 22 weeks gestation using 18- and 19-gauge needles. No foetal losses occurred, compared to a rate of foetal loss of 2.6% using amniocentesis [19]. As well as a requirement to demonstrate the safety of antenatal in vivo placental FNA, any potential diagnostic use for antenatal FNA placental sampling would inevitably depend on parallel developments in minimally invasive diagnostics, which may negate the need for FNA. We suggest that by far the most likely in vivo role for placental FNA is in placental research in animal models. Here, FNA cytology could be used to obtain placental samples at different time points in the same continuing pregnancy. This has many unique advantages and could play a role in treatment development as it would facilitate the periodic evaluation of placental structure as it develops and monitoring of the effects of pharmaceutical intervention. Before any such animal studies are conducted, however, equivalents of our study would need to be performed in each proposed animal model. These would be needed to establish feasibility and to document the normal cytological appearances of placental structures which vary across species.

The arrangement of nuclei in linear patterns within syncytiotrophoblast has been proposed as a basis for a distinctive pattern of pathological syncytial knot formation known as wave-like syncytial knots (WLSK) [16]. These linear arrangements are difficult to identify in routine histological sections, but this study has confirmed, in the most definitive manner yet, that they are a readily identifiable feature of normal villous syncytiotrophoblast, as visualised in cytology preparations. These cytology preparations also have the advantage of removing the possibility that the observed patterns represent a sectioning artefact. There is a need to better understand the nuclear–cytoskeletal interactions and processes leading to the formation of these various spatial patterns. In particular, we need to determine whether these interactions have a role in controlling syncytiotrophoblast function and turnover. If we do this, novel investigative opportunities may emerge for the investigation of syncytiotrophoblast function from the earliest stages of pregnancy. For example, in Figure 4B, the syncytiotrophoblast nuclei showing the highest degrees of nuclear chromatin condensation are seen clustered together within a longer line of nuclei. Based on this appearance, it could be hypothesised that a form of a “nuclear conveyor belt” may exist that controls this nuclear condensation process and thus syncytiotrophoblast turnover. Importantly, novel methods may also emerge for the investigation of the potential role of the processes leading to these nuclear arrangements in leading pregnancy complications, including IUGR and preeclampsia.

The variety of material obtained using the cytology methods we have used, from cytoplasm-free nuclear aggregates to flat trophoblast sheets to large microbiopsies, also show that cytology sampling has the potential to be a valuable investigative tool in future research studies of trophoblast and villous structure in both normal placental development and pathological placental processes. Some pathological conditions such as delayed villous maturation/distal villous immaturity or distal villous hypoplasia involve diffuse abnormalities of villous structural development [20,21]. Such structural anomalies may be amenable to analysis in microbiopsies obtained by FNA sampling of delivered placentas. Other placental pathologies have been linked to abnormalities in trophoblast [9,10,11,12,13,14,16]. Cytology preparations provide a new way of looking at villous trophoblast as the trophoblast sheets obtained are not obtainable in routine histology and visualisation of trophoblast nuclear morphology is superior. Documentation of (a) the normal cytological appearances of the human placenta and (b) the capability of cytology techniques to visualise the microscopic structure of the placenta in a novel way are both important first steps. Having established both, studies using similar techniques can now be embarked on to investigate pathological processes in the placenta. In these circumstances, the examination of freshly delivered human placentas from abnormal and control groups using cytology techniques would provide an entirely novel but accessible and low-cost method of investigating these conditions that may provide new insights.

## 5. Conclusions

In conclusion, we have documented for the first time the cytological appearances of the normal term human placenta. We have also shown that cytology preparations taken from delivered placentas (a) provide technically excellent material for cytological evaluation, (b) confirm the presence of complex nuclear organisational patterns in the syncytiotrophoblast, including linear nuclear arrangements, and (c) provide superior nuclear detail over standard histological sections. Finally, we have demonstrated that cytology techniques, by providing an entirely novel but accessible way of looking at the microscopic structure of the placenta, may be an untapped investigative resource for understanding placental development and growth and also placental disease. In part, this is because of its ability to both sample the freshly delivered placenta and potentially sample the placenta in vivo in animal models. By showing a proof of concept and by beginning to document the normal cytological appearance of the human placenta, this study completes a necessary first step before specific studies looking at either normal development or pathological processes using cytology techniques can be undertaken.

## Data Availability

Original cytology slides generated for this project are held in the Pathology Department of Cork University Hospital.
